# An Effective Health System Approach to End TB: Implementing the Double X Strategy in Vietnam

**DOI:** 10.9745/GHSP-D-24-00024

**Published:** 2024-06-27

**Authors:** Anh L. Innes, Victoria Lebrun, Gia Linh Hoang, Andres Martinez, Nhi Dinh, Thi Thuy Ha Nguyen, Tan Phat Huynh, Van Luong Quach, Thanh Binh Nguyen, Van Chinh Trieu, Nghi Do Bao Tran, Huy Minh Pham, Van Luong Dinh, Binh Hoa Nguyen, Thi Thanh Huyen Truong, Van Cu Nguyen, Viet Nhung Nguyen, Thu Hien Mai

**Affiliations:** a FHI 360 Asia Pacific Regional Office, Bangkok, Thailand.; b FHI 360 Vietnam, Hanoi, Vietnam.; c FHI 360, Durham, NC, USA.; d U.S. Agency for International Development/Vietnam, Hanoi, Vietnam.; e Vietnam National Lung Hospital, Hanoi, Vietnam.; f Pulmonology Department, University of Medicine and Pharmacy, Vietnam National University, Hanoi, Vietnam.

## Abstract

Countries that are high burden for TB must reverse the COVID-19 pandemic’s devastating effects to accelerate progress toward ending TB. Vietnam’s Double X (2X) strategy uses chest radiography (CXR) and GeneXpert (Xpert) rapid diagnostic testing to improve early detection of TB disease. Household contacts and vulnerable populations (e.g., individuals aged 60 years and older, smokers, diabetics, those with alcohol use disorders, and those previously treated for TB) with and without TB symptoms were screened in community campaigns using CXRs, followed by Xpert for those with a positive screen. In public non-TB district facilities, diabetics, respiratory outpatients, inpatients with lung disease, and other vulnerable populations underwent 2X evaluation. During COVID-19 restrictions in Vietnam, the 2X strategy improved access to TB services by decentralization to commune health stations, the lowest level of the health system, and enabling self-screening using a quick response mobile application. The number needed to screen (NNS) with CXRs to diagnose 1 person with TB disease was calculated for all 2X models and showed the highest yield among self-screeners (11 NNS with CXR), high yield for vulnerable populations in communities (60 NNS) and facilities (19 NNS), and moderately high yield for household contacts in community campaigns (154 NNS). Computer-aided diagnosis for CXRs was incorporated into community and facility implementation and improved physicians’ CXR interpretations and Xpert referral decisions. Integration of TB infection and TB disease evaluation increased eligibility for TB preventive treatment among household contacts, a major challenge during implementation. The 2X strategy increased the rational use of Xpert, employing a health system-wide approach that reached vulnerable populations with and without TB symptoms in communities and facilities for early detection of TB disease. This strategy was effectively adapted to different levels of the health system during COVID-19 restrictions and contributed to post-pandemic TB recovery in Vietnam.

## Abstract (precis)

This article describes an effective health system strategy to detect TB disease in Vietnam that prioritizes chest radiography and GeneXpert rapid diagnostic testing, improving access to TB services during the COVID-19 pandemic for vulnerable populations in communities and facilities.

## Teaser

**Key Findings:**

- Vietnam’s Double X (2X) strategy—using chest radiography and GeneXpert rapid diagnostic testing to improve early detection of TB disease—detected TB disease with high yield, effectively reaching TB-vulnerable populations with and without symptoms in communities and facilities.
- The 2X strategy was effectively adapted during the COVID-19 pandemic restrictions by decentralizing expertise for identifying vulnerable populations at the community level and mainstreaming TB services to non-TB district-level facilities.
- Interventions to address implementation challenges included computer-aided diagnosis to interpret chest radiographs, which improved the quality of referral for diagnostic testing. Integration of TB infection with TB disease evaluation increased household contacts’ eligibility for TB preventive treatment, a major challenge during implementation.

**Key Implications:**

- Program managers should consider using 2X to operationalize universal access to a World Health Organization-recommended rapid diagnostic as the first test for individuals with presumed TB.
- Implementers should consider evaluating vulnerable populations across the TB spectrum—from infection to asymptomatic and symptomatic TB disease—using the 2X framework to prevent, diagnose, and cure TB.

## INTRODUCTION

Countries with a high burden of TB experienced large declines in TB notifications in 2020 and 2021, corresponding with COVID-19 pandemic waves and mitigation measures.^1^ In 2022, global targets for treating TB disease and infection set at the first United Nations High-Level Meeting on TB were not reached,^2^ and 29% of people who became ill with TB were “missing” and not diagnosed or notified.^3^ To meet the 2023 High-Level Meeting commitments, countries must race to increase TB diagnosis, treatment, and prevention. Over several decades, systematic screening for TB disease has ranged from mass screening to symptom-based evaluation.^4^^–^^6^ Recent studies highlight more efficient screening algorithms and health system approaches to minimize losses along the TB cascade, as well as ensuring access to services by all TB-vulnerable populations.^7^^–^^9^ Active case-finding (ACF) is systematic, usually community-based, screening aiming to reach individuals early before presentation to health facilities,^10^ while intensified case-finding (ICF) in facilities improves health staff capacity for identifying people with presumed TB.^11^

Systematic screening should prioritize TB prevalence and the level of risk in vulnerable populations.^12^^,^^13^ The World Health Organization (WHO) strongly recommends systematic screening for household contacts and conditionally recommends systematic screening for people with TB clinical risks who are seeking care or are in care where TB prevalence is at least 100 per 100,000.^12^ Diabetics (particularly those with poorly controlled glucose),^14^ smokers,^15^ those with alcohol use disorders,^16^ and those previously treated for TB^17^ are at increased risk for TB disease. ACF tools facilitate risk prioritization,^18^^,^^19^ and studies have evaluated case-finding impact on TB notification.^20^^–^^24^

TB diagnostic algorithms may include symptom screening and chest radiography (CXR) followed by confirmatory testing, ideally with WHO-recommended rapid diagnostics (WRD). The 2023 WHO standard for universal access to WRDs defines benchmarks to improve access to and use of WRDs as the initial diagnostic test for people with presumed TB.^25^ Insufficient WRD access and utilization contribute to the global gap in TB treatment coverage.^26^ Digital CXR has regained favor as a screening and triage tool combined with a WRD.^27^ When used to refer people for GeneXpert (Xpert) (Cepheid, Sunnyvale, CA, USA) testing, CXRs can reduce the number of Xpert tests needed^28^ and improve affordability.^29^

Insufficient WRD access and utilization contribute to the global gap in TB treatment coverage.

One of WHO’s 30 high-TB-burden countries, Vietnam had an estimated 59% TB treatment coverage in 2022.^30^ The Vietnam National TB Program (NTP) has accelerated TB case-finding with the Double X (2X) strategy that uses CXR to triage Xpert testing, which was first evaluated in research studies in Vietnam starting in 2017.^31^^,^^32^ This article describes programmatic experience with implementing a comprehensive 2X TB case-finding strategy that started in March 2020, the challenges and adaptations made during the first 3 years of implementation, and lessons learned for the future.

## DOUBLE X STRATEGY FOR TB CASE-FINDING

### Setting

From March 2020 through December 2022, the U.S. Agency for International Development (USAID) Support to End TB project conducted 2X programmatic activities in 9 provinces (Supplement Table S1), which included 1 Northern, 2 Central, and 6 Southern provinces in Vietnam. In terms of TB prevalence, Vietnam’s 3 regions vary, with the highest TB prevalence in the South.^33^^,^^34^ Within each province, districts were conveniently selected and added each year (2020–2022) based on consultation with NTP and provincial officials.

### Double X Case-Finding for Community and Health Facility Settings

ICF facility-based implementation started in 19 districts, with annual mobile ACF campaigns in a subset of these districts. Each year, ICF expanded to additional districts, and ACF sites changed to maximize geographic coverage. Facility-based ICF integrated TB evaluation with other health services in provincial and district-level public facilities, which were predominantly outside of the NTP system.

ACF campaigns used mobile CXR vans to screen household contacts who lived, slept (1 night/week), or stayed (1 hour/day for 5 weeks) in the same house for 3 months with an index patient before diagnosis. Index patients were adults with pulmonary TB disease who were diagnosed with bacteriologically or clinically confirmed TB within 2 years of the campaign. ACF campaigns also evaluated other TB-vulnerable populations, who were elderly (aged 60 years and older), diabetics, smokers (any smoking history), regular alcohol users (daily), or malnourished (low body mass index), as well as those with pulmonary or chronic diseases, prior treatment for TB disease, or living with HIV. Risk factors for TB and comorbid illnesses were self-reported. Individuals without a defined risk for TB but reporting respiratory symptoms were also eligible for 2X ACF evaluation. NTP staff conducted home visits to identify and invite household contacts to the 2X campaigns; other TB-vulnerable populations were invited to participate through community volunteers and radio announcements. TB symptoms (fever, cough of any duration, weight loss, and night sweats) were documented but were not required to receive a CXR among household contacts and those with defined TB risks. For TB-presumptive CXRs, sputum specimens were immediately collected for Xpert testing (Xpert MTB/RIF or Xpert Ultra). Provincial physicians also ordered Xpert tests for participants with normal CXRs who screened positive for symptoms.

**FIGURE 1:**
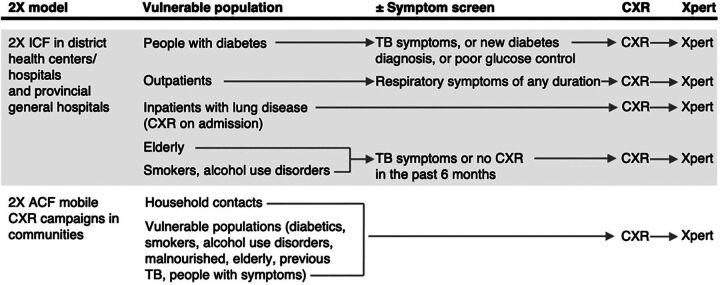
The 2X Health System Strategy to Screen and Diagnose TB Disease,^a^ Vietnam Abbreviations: 2X, Double X; ACF, active case-finding; CXR, chest radiography; ICF, intensified case-finding; Xpert, GeneXpert; ±, with or without. ^a^ Facility-based ICF and community ACF reached a range of vulnerable populations, who were evaluated (with or without symptoms) followed by CXRs, and, if TB-presumptive, Xpert testing.

In provincial and district general (non-TB) facilities, 2X ICF evaluated the following populations: (1) diabetes outpatients who were newly diagnosed, had elevated hemoglobin A1C or random blood glucose, or TB symptoms; (2) inpatients with lung disease (without specific symptom criteria, since it was assumed that inpatients would be symptomatic) and outpatients in general medical clinics with respiratory symptoms of any duration. In 2022, 2X expanded to all individuals receiving CXRs in each facility. (3) Individuals aged 60 years and older, smokers (10 or more cigarettes per day), and regular alcohol users (6 servings or more at a time, weekly for 3 months) were identified using a questionnaire in a subset of facilities and underwent 2X evaluation if they had TB symptoms or no recent CXR within 6 months. The 2X ICF algorithm adjusted screening criteria by each vulnerable population, followed by CXR; those with TB-presumptive CXRs were referred for Xpert testing (Figure 1).

### Chest Radiography Interpretation

Mobile CXR vans obtained posterior-anterior digital CXR images (Vikomed, Hanoi, Vietnam) during ACF campaigns, which were immediately interpreted on site by provincial radiologists who had access to participants’ names, ages, and a brief medical history. CXR images were interpreted as TB-presumptive or TB-negative. For 2X ICF in health facilities, CXR images were interpreted by an on-site physician, who was either a radiologist or clinician (diabetes, general, or TB physician).

### Integration of Diagnostic Testing for TB Infection

**FIGURE 2:**
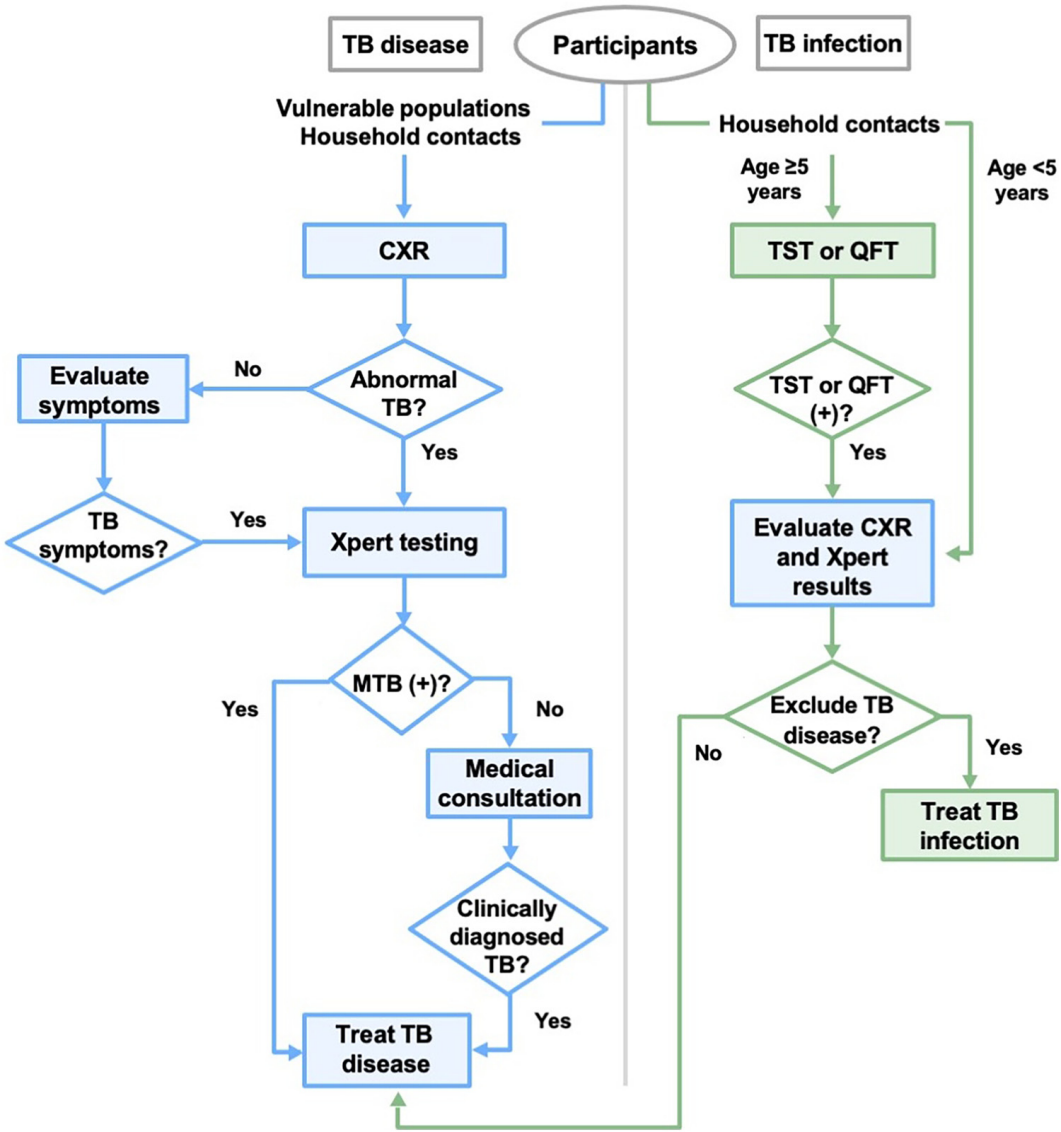
Algorithms for TB Disease and TB Infection Among Household Contacts, Vietnam Abbreviations: CXR, chest radiography, MTB, Mycobacterium TB; TST, tuberculin skin test; QFT, QuantiFERON-TB Gold Plus; Xpert, GeneXpert; (+), positive diagnostic test result.

During 2X ACF campaigns, household contacts of individuals with drug-susceptible pulmonary TB disease were evaluated for TB infection with tuberculin skin tests (TST) (Figure 2), which has been previously described.^35^ A subset of individuals (2,887 [8.8%]) received both QuantiFERON TB-Gold Plus (QFT) (Qiagen, Hilden, Germany) and TST for quality assurance. Simultaneous evaluation for TB disease and TB infection capitalized on the “1-stop shop” ACF campaigns to screen for both TB disease and infection in a single encounter.

### Data Sources

ACF and ICF data comprising the TB disease and TB infection diagnostic cascades, sex, and age were collected by health care staff, consolidated into monthly reports, and submitted to project staff. Individualized sex and age data were collected for each ACF participant; aggregated sex and age were collected for ICF implementation. Monthly reports were entered into a Power BI (Microsoft, Seattle, Washington, USA) online database. Project costs to implement 2X were calculated from direct costs paid by the project for health staff labor, consumables (Xpert testing), and CXRs.

### Ethical Approval

This study was conducted as public health programming that did not require or receive ethics review. Participants provided verbal consent before participation.

## PROGRAM RESULTS

### Double X TB Disease and TB Infection Cascade Results

2X ACF campaigns screened 21,529 household contacts with CXRs, of which 9.8% were TB-presumptive, leading to 2,217 Xpert tests and 140 people diagnosed with all forms of TB disease (138 Xpert-confirmed) (Table 1). CXRs were obtained for 79,051 TB-vulnerable populations during 2X ACF, resulting in 14,077 (17.8%) TB-presumptive CXRs and 1,255 (9.1%) individuals with Xpert-confirmed TB disease. The number of CXRs needed for 1 TB diagnosis (NNS) was lower for ACF TB-vulnerable populations (60 NNS with CXR) than ACF household contacts (154 NNS with CXR), although the detection yield of 650 TB cases per 100,000 CXRs was still 3.7 times the 2022 Vietnam national TB incidence rate of 176 per 100,000 population.^30^ Facility-based 2X ICF evaluated a total of 573,944 people with CXRs, and rates for TB-presumptive CXRs varied widely by ICF population. Xpert positivity for 2X ICF populations was higher overall than for ACF populations. We found the highest ICF TB yield for the composite group of smokers, those with alcohol use disorders, and elderly individuals (19 NNS with CXR); followed by respiratory inpatients and outpatients (92 NNS with CXR); and diabetes outpatients (100 NNS with CXR). Linkage to TB treatment was lower overall for 2X ACF than ICF. Clinically diagnosed TB (Xpert-negative with TB-presumptive CXR) was likely higher than reported due to some inconsistencies in how sites aggregated ACF and ICF data. Across all years of ACF implementation, rates for TB-presumptive CXRs and TB detection yield were higher in males than females and higher for older age groups (Table 2).

**TABLE 1.** TB Cascade for 2X ACF and ICF Implementation, March 2020–December 2022, Vietnam

**Model**	**Screened With CXRs, No.**	**TB-Presumptive CXRs, No. (%)**	**Tested With Xpert, No.**	**Xpert Positive, No. (%)**	**TB Diagnosed (All Forms), No.**	**All Forms TB Yield per 100,000 CXR, No.**	**NNS With CXR**	**Cost to Detect 1 Person With TB Disease, US$**	**Initiated on TB Treatment, No. (%)**
2X ACF community campaigns	100,580	16,197 (16.1)	16,033	1,393 (8.7)	1,448	1,440	69		1,193 (82.4)
Household contacts	21,529	2,120 (9.8)	2,217	138 (6.2)	140	650	154	739	121 (86.4)
Other TB vulnerable populations	79,051	14,077 (17.8)	13,816	1,255 (9.1)	1,308	1,655	60		1,072 (82.0)
2X ICF in health facilities	573,944	30,688 (5.3)	28,676	6,375 (22.2)	6,465	1,126	89	83	5,818 (90.0)
Diabetes outpatients	73,025	5,324 (7.3)	5,027	724 (14.4)	730	1,000	100	446	667 (91.4)
Smokers, alcohol use disorders, elderly^a^	6,539	1,703 (26.0)	1,605	328 (20.4)	338	5,169	19	116	304 (89.9)
Inpatients and outpatients	494,380	23,661 (4.8)	22,044	5,323 (24.1)	5,397	1,092	92	31^b^	4,847 (89.8)

Abbreviations: 2X, Double X; ACF, active case-finding; CXR, chest radiography; ICF, intensified case-finding; NNS, number needed to screen; Xpert, GeneXpert.

^a^ ICF smokers, those with alcohol use disorders, and those aged 60 years and older were evaluated in Dong Thap and Thai Binh provinces in 2021, and in An Giang province in 2022.

^b^ CXR costs were covered by the project for ACF and all ICF populations except inpatients and outpatients with clinical indications for TB whose CXRs were covered by social health insurance. Supplement Table S2 lists full cost details for all models.

**TABLE 2.** CXR and Xpert Results by Demographic Characteristics for 2X ACF and ICF Participants, March 2020–December 2022, Vietnam

** **	**CXRs, No.**	**TB-Presumptive CXRs, No. (%)**	**Tested With Xpert, No.**	**Xpert Positive, No. (%)**	**TB Diagnosed (All forms), No.**	**All Forms TB Yield per 100,000 CXR, No.**	**NNS With CXR**
ACF	100,580	16,197 (16.1)	16,033	1,393 (8.7)	1,448	1,440	69
Age, years							
<5	693	94 (13.6)	181	3 (1.7)	3	433	231
5–14	4,481	263 (5.9)	288	4 (1.4)	4	89	1,120
15–24	2,968	221 (7.4)	213	18 (8.5)	18	606	165
25–34	5,346	499 (9.3)	491	47 (9.6)	47	879	114
35–44	10,367	1,109 (10.7)	1,073	99 (9.2)	101	974	103
45–54	17,173	2,419 (14.1)	2,391	227 (9.5)	234	1,363	73
55–64	26,546	4,521 (17)	4,453	393 (8.8)	421	1,586	63
≥65	30,880	7,004 (22.7)	6,918	515 (7.4)	533	1,726	58
Sex							
Female	56,208	5,292 (9.4)	5,284	269 (5.1)	282	502	199
Male	44,372	10,905 (24.6)	10,749	1,124 (10.5)	1,166	2,628	38
ICF	573,944	30,688 (5.3)	28,676	6,375 (22.2)	6,465	1,126	89
Age, years^a^							
<5	NA	NA	495	13 (2.6)	15	NA	NA
5–14	NA	NA	269	35 (13.0)	40	NA	NA
15–24	NA	NA	536	225 (42.0)	227	NA	NA
25–34	NA	NA	1,249	461 (36.9)	464	NA	NA
35–44	NA	NA	2,359	838 (35.5)	844	NA	NA
45–54	NA	NA	4,346	1,317 (30.3)	1,331	NA	NA
55–64	NA	NA	7,262	1,571 (21.6)	1,593	NA	NA
≥65	NA	NA	11,003	1,658 (15.1)	1,694	NA	NA
Age NA	NA	NA	1,157	257 (22.2)	257	NA	NA
Sex							
Female	279,292	11,000 (3.9)	10,272	1,450 (14.1)	1,475	528	189
Male	294,652	19,688 (6.7)	18,404	4,925 (26.8)	4,990	1,694	59

Abbreviations: ACF, active case-finding; CXR, chest radiography; ICF, intensified case-finding; NA, not available; NNS, number needed to screen; Xpert, GeneXpert.

^a^ CXRs disaggregated by age groups were not available for ICF implementation.

Project costs for 2X ACF and ICF to detect 1 person with TB disease are shown in Table 1 and Supplement Table S2, which exclude costs paid by the Global Fund and national social health insurance. Xpert cartridges were provided in-kind by the NTP under Global Fund support. Diagnostic costs for labor and consumables were covered by the project for all models. CXR costs were covered by the project for ACF and all ICF populations except ICF inpatients and outpatients with clinical indications for TB, whose CXRs were covered by national social health insurance. For all models combined, the project’s approximate annual costs for training (US$102,000) and supervision (US$22,000) were in addition to the cost per case.

From March 2020 through December 2022, of the 32,712 individuals tested for TB infection, 2X-integrated TB infection testing evaluated 32,494 individuals, of whom 5,126 (15.7%) were positive; 4,986 were eligible for TB Preventive Treatment (TPT) after excluding TB disease, and 3,171 (63.6%) initiated TPT. To our knowledge, this was the first programmatic implementation in Vietnam of a comprehensive strategy for TB disease detection in both community and facility settings that integrated TB infection diagnosis for household contacts of all ages. Programmatic TPT (without TB infection diagnosis) had previously been provided in Vietnam for people living with HIV and child contacts aged younger than 5 years.

To our knowledge, this was the first programmatic implementation in Vietnam of a comprehensive strategy for TB disease detection in community and facility settings that integrated TB infection diagnosis for household contacts of all ages.

## KEY IMPLEMENTATION CHALLENGES AND SOLUTIONS

### Limited Access to TB Services Due to COVID-19 Pandemic Restrictions

COVID-19 pandemic lockdowns in 2020–2021 halted ACF and severely impacted ICF implementation. A consultative process with the NTP identified and prioritized solutions, requiring adaptations of the original 2X strategy. To reach household contacts independent of ACF community campaigns, a hybrid ACF/ICF model comprised active outreach by phone followed by 2X evaluation for TB disease and TB infection in nearby facilities. To overcome barriers to facility-based 2X ICF, 2 models were implemented at the community level. First, 2X was adapted for commune health stations—the most decentralized level of the health system where there is no CXR—to screen people for cough longer than 2 weeks followed by referral for Xpert. CXR was not included in this “Single X” (Xpert-only) algorithm. Self-screening using a quick-response (QR) code mobile application also made TB screening available to anyone with a smartphone or tablet. In 2022, it was estimated that 79% of the Vietnamese population use the Internet.^36^ QR code self-screening evaluated people for symptoms and TB risk factors and referred those with positive screens to the nearest facility for 2X evaluation.

These 2X models adapted during COVID-19 restrictions evaluated a total of 20,050 individuals (Table 3). The hybrid ACF/ICF model evaluated 8,674 household contacts with CXRs, resulting in a higher CXR TB-presumptive rate (16.9.%) and TB yield (90 NNS with CXR) than household contacts in ACF community campaigns (154 NNS with CXR) (Table 1). The Single X model referred 8,822 people for Xpert testing, leading to 954 (10.8%) people with Xpert-confirmed TB disease. The QR-code self-screening model led to 2,554 people receiving CXRs, among whom 41.6% had TB-presumptive CXRs, detecting 228 people with TB disease (217 [20.7%] Xpert-confirmed) (Table 3). This was the highest yield among all models (11 NNS with CXR). Detailed cost data for the adapted 2X models are shown in Supplement Table S2.

**Figure:**
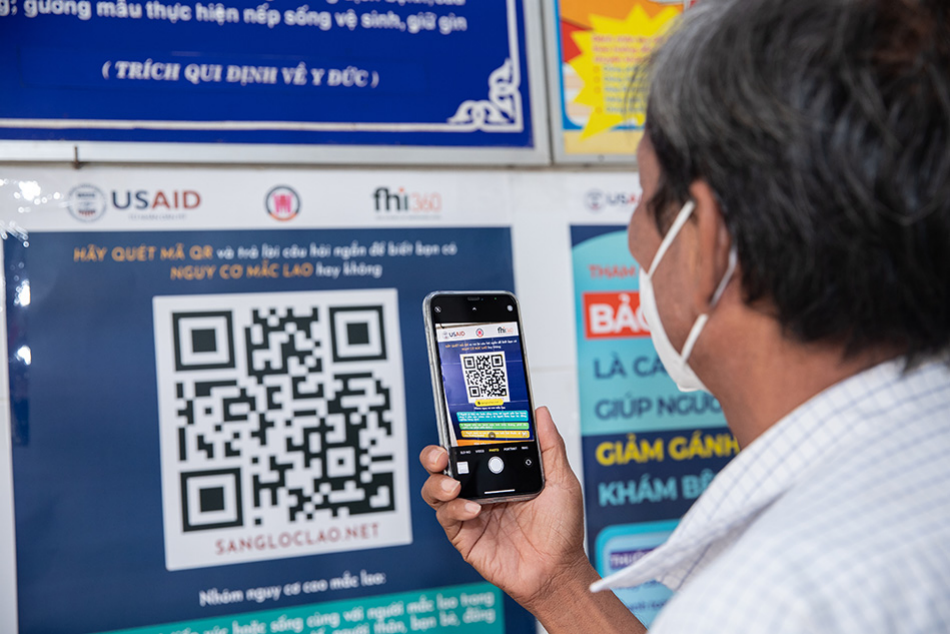
A man scans the quick-response code to complete a TB screening questionnaire in Tay Ninh province, 2022. © 2022 Vu Ngoc Dung/FHI 360

**TABLE 3.** TB Cascade for the 2X Strategy Adapted During the COVID-19 Pandemic, March 2021–December 2022, Vietnam

**2X Model**	**Screened With CXRs, No.**	**TB-Presumptive CXRs, No. (%)**	**Tested With Xpert, No.**	**Xpert Positive, No. (%)**	**TB Diagnosed (All Forms), No.**	**All Forms TB Yield per 100,000 CXR, No.**	**NNS With CXR**	**Cost to Detect 1 Person With TB Disease, US$**	**Initiated on TB Treatment, No. (%)**
Hybrid ACF/ICF from March 2021	8,674	1,463 (16.9)	1,575	91 (5.8)	96	1,107	90	718	84 (87.5)
Single X from October 2021	N/A	N/A	8,822	954 (10.8)	962	N/A	N/A	72	830 (86.3)
QR code self-screen from April 2022	2,554	1,062 (41.6)	1,048	217 (20.7)	228	8,927	11	225	213 (93.4)

Abbreviations: 2X, Double X; ACF, active case-finding; CXR, chest radiography; ICF, intensified case-finding; N/A, not applicable; NNS, number needed to screen; QR, quick response; Single X, GeneXpert-only algorithm.

### Low Rates of Tuberculin Skin Test Positivity and TB Preventive Treatment Initiation in Active Case-Finding Campaigns

Integrating TB infection diagnosis and TPT into 2X ACF required training to accurately identify household contacts and to build skills on TST injection and interpretation. Early implementation in 2020–2021 ACF campaigns showed low TST positivity for household contacts compared with previous research results in Vietnam,^37^ prompting the USAID Support to End TB project to explore issues with TST as previously described.^35^ Among household contacts aged 5 years and older in 2020, QFT positivity (38.6%) had higher agreement with TST ≥5 mm (37.4%) than TST ≥10 mm (13.1%); QFT results were not discordant with TST ≥5 mm but were discordant with TST ≥10 mm. Older age groups (>30 years), but not sex, increased odds for positive QFT and TST ≥5 mm. In November 2021, the NTP decreased the TST induration threshold for household contacts aged 5 years and older from ≥10 mm to ≥5 mm; the proportion diagnosed with TB infection increased from 12.6% to 26.4% (Table 4). Low uptake of TPT required sensitization of providers, families, and communities to emphasize the importance of treatment for TB infection to prevent progression to TB disease. TPT initiation improved from 54.0% to 79.4%.

**TABLE 4.** TB Infection Cascade Results Before and After Changing the TST Induration Threshold, Vietnam

	**TB Infection**^a^ **Diagnostic Tests, No.**	**TB Infection Test Results, No. (%)**	**Positive TB Infection Test Results, No. (%)**	**Eligible for TPT After Excluding TB Disease, No.**	**Initiated on TPT, No. (%)**
March 2020–October 2021: TST threshold ≥10 mm					
ACF community campaigns	25,378	25,320 (99.8)	3,190 (12.6)	3,101	1,674 (54.0)
November 2021–December 2022: TST threshold ≥5 mm					
ACF community campaigns	7,334	7,174 (97.8)	1,936 (26.4)	1,885	1,497 (79.4)

Abbreviations: ACF, active case-finding; QFT, QuantiFERON-TB Gold Plus; TPT, TB preventive treatment; TST, tuberculin skin testing.

^a^ TB infection was diagnosed using TST; in a subset of individuals (2,887 [8.8%]) who received both TST and QFT for quality assurance, a positive TST or QFT result yielded a diagnosis of TB infection. COVID-19 pandemic restrictions decreased the number undergoing TB infection evaluation in 2022.

Integrating TB infection diagnosis and TPT into 2X ACF required training to accurately identify household contacts and to build skills on TST injection and interpretation.

### Variable Quality of Chest Radiography Interpretation

**FIGURE 3:**
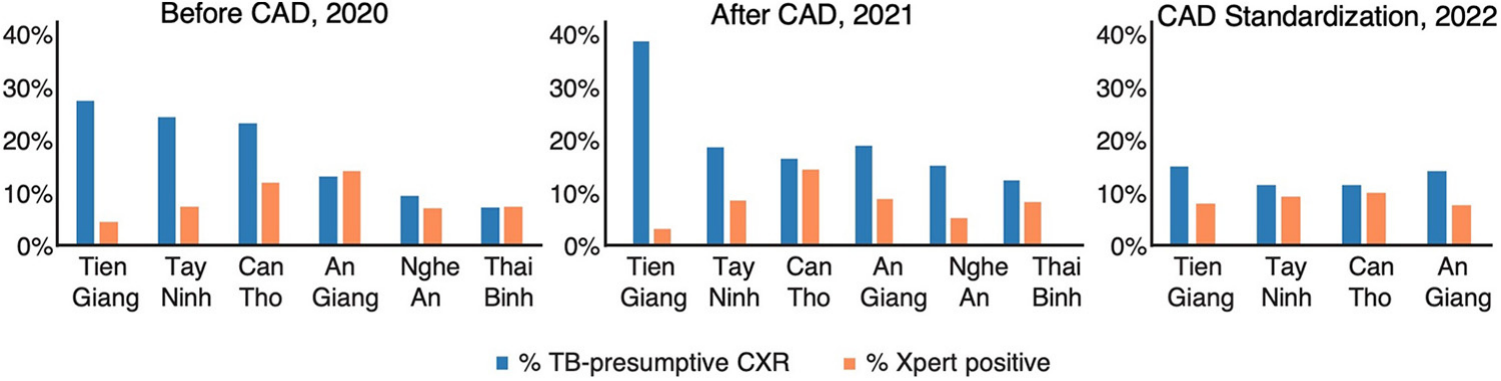
Rates for TB-Presumptive CXR and Xpert Positivity From 2X ACF Before CAD, After CAD Piloting, and CAD Standardization^a^ Abbreviations: 2X, Double X; ACF, active case-finding; CAD, computer-aided diagnosis; CXR, chest radiography; Xpert, GeneXpert. ^a^ Number of CXRs interpreted for each year: 2020, N=52,868; 2021, N=19,275; and 2022, N=28,437.

CXRs are more sensitive than specific for detecting pulmonary TB disease and have known inter- and intra-reader variability.^38^ In 2020 ACF campaigns, the proportion of TB-presumptive CXRs varied widely across provinces, with a range of Xpert positivity rates (Figure 3). To improve the quality of CXR interpretation, computer-aided diagnosis of CXRs (CAD) was integrated into 2X ACF using qXR 3.0 (Qure.ai, Mumbai, India). CAD was piloted starting in 2021 for ACF campaigns using different models for integration. In 2022, CAD integration was standardized using the “CAD-first” model, in which only CXRs with CAD scores above the TB threshold (qXR ≥0.4) were read by radiologists. Following CAD-first integration, there was a trend toward decreased rates of TB-presumptive CXRs in 2022 (Figure 3), most notably for Tien Giang Province that had very high rates of TB-presumptive CXRs in 2020 (27%) and 2021 (39%) resulting in high referral rates for Xpert testing. The quality of CXR interpretation in Tien Giang improved with CAD-first integration in 2022, decreasing the rate of TB-presumptive CXRs (17%) and increasing Xpert positivity from 4% (2020) and 3% (2021) to 9% (2022).

CAD integration into the 2X workflow was smooth for ACF campaigns because they focused solely on TB evaluation, while CAD facility integration required more intensive efforts. Starting in 2022, CAD was piloted in 7 district-level facilities, integrated in parallel with physician interpretation (both CAD and physicians read all CXRs) using the CAD TB threshold, qXR ≥0.60, as previously described.^39^ District facility radiologists were trained to select CXRs for CAD interpretation targeting individuals undergoing TB triage for symptoms or risks (not TB treatment follow-up), and to coordinate Xpert referral for those with TB-presumptive CXRs. A training handbook was developed and implemented to identify TB abnormalities on CXRs. Comparing the TB diagnostic cascade before (2021) and after (2022) CAD facility integration, we found an increase in the rate of TB-presumptive CXRs (10.7% to 19.9%) and TB detection yield (3,538 to 4,645 per 100,000 CXRs) after CAD integration (Table 5). In 2022, CAD facilities also outperformed non-CAD facilities in the same provinces, which had lower rates of TB-presumptive CXRs (8.9%) and TB detection yield (2,357 per 100,000 CXRs) than CAD facilities. In 2022 post-CAD implementation, the proportion initiating treatment was lower for both CAD (91.1%) and non-CAD (90.3%) facilities than pre-CAD (97.0%); this was due to multiple factors including the rapid scale-up of 2X ICF in all sites (CAD and non-CAD) in the context of pandemic recovery in 2022. Overall, CAD integration improved the quality of CXR interpretation and referral for Xpert testing for both ACF and ICF implementation.

**TABLE 5.** TB Cascade Before and After CAD Integration Into 2X ICF Facility Implementation, Vietnam

	**Evaluated With CXRs, No.**	**TB-Presumptive CXRs, No. (%) **	**Tested With Xpert, No. (%)**	**TB Diagnosed (All Forms), No. (%)**	**All Forms TB Yield/ 100,000 CXR, No.**	**Initiated on TB Treatment, No. (%)**
Before CAD: April–December 2021						
CAD facilities (n=7)	12,351	1,322 (10.7)	1,320 (99.8)	437 (33.1)	3,538	424 (97.0)
After CAD: April–December 2022						
CAD facilities (n=7)	19,289	3,884 (19.9)	3,352 (85.9)	896 (26.7)	4,645	816 (91.1)
Non-CAD facilities (n=23)	81,245	7,269 (8.9)	7,029 (96.4)	1,915 (27.2)	2,357	1,729 (90.3)

Abbreviations: CAD, computer-aided diagnosis; CXR, chest radiograph; ICF, intensified case-finding; Xpert, GeneXpert.

### Challenges With GeneXpert Utilization

2X implementation required providers to use Xpert as the first TB diagnostic test for all people with TB-presumptive CXRs, aiming to replace sputum smear microscopy, which was still widely used. Barriers included low-quality sputum samples and sample processing and transportation to Xpert sites. Standard operating procedures and job aids complementing training and on-site technical assistance (TA) addressed the perception that Xpert testing is challenging. Before the project, health staff in non-TB facilities’ outpatient departments and diabetes clinics had very limited experience with routine triage and testing for TB disease; tackling this required ongoing advocacy with facility leaders to effectively prioritize TB evaluation within non-TB departments. Health staff training was also critical for effective CAD integration into CXR evaluation for 2X ICF because this required coordination among multiple departments—physicians ordering the CXR (e.g., diabetes); reading the CXR (e.g., radiology); and ordering Xpert tests (TB unit).

**FIGURE 4:**
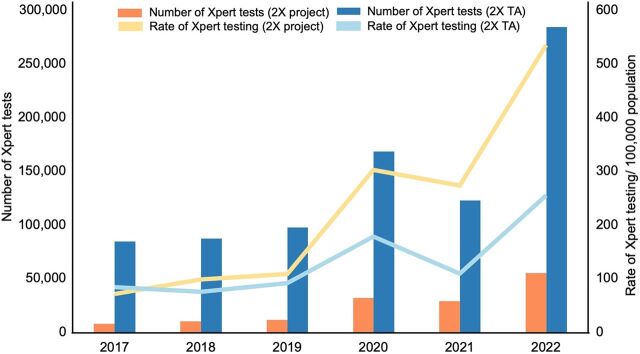
Xpert Testing for Project Provinces Directly Implementing 2X^a^ Compared to Non-Project Provinces Receiving TA on 2X Abbreviations: 2X, Double X; TA, technical assistance; Xpert, GeneXpert. ^a^ For 2X project sites, data were analyzed from 6 provinces that implemented for the full duration from 2020 through 2022, excluding 3 provinces that phased in or out of direct implementation. Data before project implementation (2017–2019) are shown for comparison with project results (2020–2022).

In October 2020, the NTP approved the project’s 2X standard operating procedure as the national guideline, expanding to additional provinces that implemented the guideline within their capacity and resources. Provinces that did not implement directly under the USAID project received TA and health staff training. To assess Xpert utilization in project provinces and TA provinces, aggregated Xpert testing data from the National TB Reference Laboratory were analyzed and showed that population-adjusted Xpert testing rates from 2020 to 2022 appeared to increase faster in project provinces than TA-only provinces (Figure 4); 2017–2019 data are included to show Xpert testing trends before project implementation.

## LESSONS LEARNED

Access to accurate diagnostic testing is critical to detect TB.^7^^,^^9^ Despite implementation challenges, Vietnam’s 2X strategy detected TB disease with high yield, effectively evaluated TB-vulnerable populations in facility and community settings, and integrated TB infection with TB disease for household contacts. The initial 2X ACF and ICF algorithms were successfully adapted to expand access to TB services during the COVID-19 pandemic, which not only mitigated health system disruptions but also leveraged existing resources, improved people-centeredness by reaching vulnerable populations where they are, and decentralized TB expertise to the community level.

Vietnam’s 2X strategy detected TB disease with high yield, effectively evaluated TB-vulnerable populations in facility and community settings, and integrated TB infection with TB disease for household contacts.

### Decentralizing TB services to the Community Level Improves Access for Early Detection

When 2X ACF campaigns could not be conducted during pandemic restrictions, the “hybrid” ACF/ICF model enabled active outreach to household contacts, simultaneously building health staff capacity for routine TB contact investigation and improving follow-up of household contacts who did not attend the ACF campaigns. Two other approaches brought screening to vulnerable populations independent of ACF campaigns. The commune health station is the most decentralized level of the health system, and 2X was adapted into Single X to enable people with symptoms to be evaluated in a facility close to their home. Training on Single X reinforced TB screening principles among community-level health providers and expanded the health workforce capable of ordering Xpert tests, task-shifting from district and provincial-level facilities. QR code self-screening reached individuals anywhere in the community and resulted in the highest yield among self-screeners who completed a referral; these individuals often sought care because of symptoms. We learned that online self-screening can be easily rolled out to reach symptomatic people, provide a referral, and collect contact information, enabling the district TB units to follow up.

### Engaging Non-TB Public Facilities Captures Vulnerable Populations Seeking Care

During the COVID-19 pandemic, when the majority of NTP facilities were diverted from TB services, 2X ICF engaged non-TB public facilities and expanded TB screening in these facilities to all individuals with CXRs, regardless of their reason for presenting to care. A key benefit of expanding ICF to non-TB public facilities was the opportunity to strengthen TB triage skills and technical capacity, mainstreaming TB services to general facilities. Effective and efficient diagnostic algorithms such as 2X enable timely evaluation for individuals with TB risks or symptoms; this can address incomplete follow-through across the TB cascade that is a known challenge in many settings.^8^

### Integrating TB Infection With TB Disease Evaluation Leverages Resources and Improves TPT Uptake

Our experience shows that it is feasible to integrate TB infection and TB disease detection and when conducted simultaneously, the integrated approach leverages commonalities (e.g., CXR and symptom screening) to save time and costs. Despite significant challenges, TST remains the most pragmatic option to diagnose TB infection. Newer diagnostic methods, such as interferon gamma release assays^40^ and antigen-based skin testing,^41^ are more specific than TST but have their own limitations with scalability and cost. Modeling studies show that ending TB requires treatment of both TB disease and TB infection (Vietnam National TB Strategic Plan 2021–2026); high-TB-burden countries thus need to determine TPT eligibility, which can be challenging. Integration of TB infection with TB disease also enables coverage of the TB spectrum, from TB infection to asymptomatic (subclinical) and symptomatic TB disease. An effective strategy to end TB should address all forms of prevalent TB, which includes subclinical and symptomatic TB disease.^42^ The comprehensive 2X strategy shows how to operationalize this approach.

The integrated TB infection and TB disease detection leverages commonalities to save time and costs.

### Limitations

This implementation study was not designed to prospectively match 2X intervention with control sites, and most implementation occurred during the COVID-19 pandemic. We are thus unable to compare TB notification from 2X sites with controls; and comparison with pre-intervention notifications is challenged by wide variability in diagnostic testing and notifications during the COVID-19 pandemic. Data were analyzed from 2X provinces to measure TB notifications over time and showed that the rate of TB notifications decreased (2021) and then increased during COVID-19 pandemic recovery (2022), but there are insufficient time points after the COVID-19 pandemic to show a sustained increase in notifications (Table 6).^43^ The 2X health system strategy is high yield for TB detection with strong performance across the TB cascade, but more data are needed to determine if this strategy will impact TB epidemiology in Vietnam.

**TABLE 6.** All Forms TB Notifications per Year in 2X Provinces,^a^ Vietnam, 2017–2022

	**All Forms TB Notified, No.**	**Rate of All Forms TB Notified per 100,000 Population**
2017	14,854	127.24
2018	14,755	125.88
2019	14,753	125.38
2020	14,688	124.39
2021	10,690	90.25
2022	15,207	128.04

Abbreviation: 2X, Double X.

^a^ Annual TB notifications at the provincial level from the National TB Program’s Vietnam TB Information Management Electronic System were used to calculate notification rates per 100,000 population, using the total TB notifications for each province by year among the provinces’ total annual populations. Estimates of subnational populations in Vietnam for 2015–2030 were obtained from the U.S. President’s Emergency Plan for AIDS Relief’s U.S. Census Bureau.^43^ Data were analyzed for the 6 2X provinces that implemented for the full duration from 2020–2022; data from pre-project implementation (2017–2019) are shown to compare trends.

We describe a programmatic implementation that was not conducted under a research protocol. These “real-world” conditions may have led to site-level differences during selection, screening, or testing individuals for TB disease or TB infection. COVID-19 restrictions significantly altered routine program conditions and may have impacted health-seeking behavior, which we have not measured or analyzed. Finally, we report the programmatic cost per case detected using direct costs paid to implement 2X within the existing public health system. Costing was not formally conducted for this report, but a cost-effectiveness analysis is currently ongoing.

## CONCLUSIONS

Our experience in Vietnam shows how to harness the potential of CXR and Xpert by implementing a health system approach that spans community and facility settings and increases access to testing for vulnerable populations with and without symptoms. The 2X strategy paves the way for universal access to WRDs in Vietnam, currently prioritizing Xpert but including other rapid diagnostics as they become available and are approved. 2X also integrates TB infection and TB disease detection for household contacts, an approach that can expand to other high-priority populations for TPT. Altogether, this strategy operationalizes a framework for evaluating TB-vulnerable populations across the TB spectrum—from infection to subclinical and symptomatic TB disease—through routine ACF and ICF implementation.

## Supplementary Material

GHSP-D-24-00024-Innes-Supplements.pdf

## Notes

**Peer Reviewed**
