# Supplementary material for: An Effective Health System Approach to End TB: Implementing the Double X Strategy in Vietnam
Source: Glob Health Sci Pract. 2024 Jun 27;12(3):e2400024. doi: 10.9745/GHSP-D-24-00024 (PMC11216706; doi:10.9745/GHSP-D-24-00024)
Supplement: GHSP-D-24-00024-Innes-Supplements.pdf [file GHSP-D-24-00024-Innes-Supplements.pdf]

Supplement to: Innes AL, Lebrun V, Hoang GL, et al. An effective health system approach to end TB: implementing the double X strategy in Vietnam. *Glob Health Sci Pract*. 2024;12(3):e2400024. https://doi.org/10.9745/GHSP-D-24-00024

Supplement Table S1. Project Provinces Implementing 2X from March 2020–December 2022.

Province	Region	Province Population,[a] No.	Rural Population, (%)	Total Districts, No.	Districts Implementing 2X, No.	2X District Population,[a] No.	Rural Population in 2X Districts, (%)
An Giang	South	1,908,352	80%	11	11	1,908,352	80%
Can Tho	South	1,235,171	34%	9	5	742,361	41%
Dong Nai	South	3,097,107	66%	9	3	1,528,113	31%
Dong Thap	South	1,599,504	77%	10	3	479,329	100%
Khanh Hoa	Central	1,231,107	47%	9	3	503,491	54%
Nghe An	Central	3,327,791	83%	21	4	1,229,514	28%
Tay Ninh	South	1,169,165	62%	9	9	1,169,165	62%
Thai Binh	North	1,860,447	89%	8	4	917,995	100%
Tien Giang	South	1,764,185	74%	11	4	709,501	82%

Abbreviations: 2X, Double X.

[a] Estimates of provincial and district populations are obtained from the General Statistics Office. Completed Results of the 2019 Vietnam Population and Housing Census – General Statistics Office. Accessed June 6, 2024. https://www.gso.gov.vn/wp-content/uploads/2019/12/Ket-qua-toan-bo-Tong-dieu-tra-dan-so-va-nha-o-2019.pdf

Supplement to: Innes AL, Lebrun V, Hoang GL, et al. An effective health system approach to end TB: implementing the double X strategy in Vietnam. *Glob Health Sci Pract*. 2024;12(3):e2400024. https://doi.org/10.9745/GHSP-D-24-00024

Supplement Table S2. Estimated Project Costs to Detect 1 Person with TB Disease for 2X ACF, ICF and COVID-Adapted Models

Model	Labor Cost[a] (US$)	CXR Cost[b] (US$)	Xpert Cost[c] (US$)	Total Cost (US$)	TB Diagnosed, No.	Cost to Detect 1 Person With TB (US$)
**ACF community campaigns**	352,204	142,659	46,699	**541,562**	733	**739[d]**
**ICF in health facilities**	105,555	219,333	131,880	**456,768**	5,505	**83**
Diabetes outpatients	55,485	199,716	20,856	**276,057**	619	**446**
Smokers, alcohol use disorders, elderly	10,555	19,617	9,032	**39,204**	338	**116**
Inpatients and outpatients	39,515	N/A	101,991	**141,506**	4,548	**31**
**Hybrid ACF/ICF**	33,950	26,139	8,869	**68,958**	96	**718[d]**
**Single X**	19,790	N/A	49,648	**69,437**	962	**72**
**QR code self-screen**	37,715	7,636	5,898	**51,250**	228	**225**

Abbreviations: 2X, Double X; ACF, active case finding; CXR, chest radiography; ICF, intensified case finding; N/A, not applicable; QR, Quick Response; Single X, GeneXpert-only; TB, tuberculosis; US$, US Dollar; Xpert, GeneXpert.

[a] Labor cost: ACF community campaigns: Support fee for health staff to reach, counsel, and invite participants to campaigns and to provide health services in campaigns (based on cost norms of the Government of Vietnam and Global Fund); print materials; rent tables and chairs; provide personal protective equipment including face masks and hand sanitizer; ICF in health facilities: Support fee for health staff of outpatient departments to screen, counsel, test and diagnose and/or link people to District TB Units; Hybrid, Single X, QR code: Support fee for health staff to reach, counsel, and invite participants and to link them to health facilities.

[b] CXR cost: $2.99/CXR (Government of Vietnam cost norm). This table shows CXR costs that are covered by the project. For 2X ICF inpatients and outpatients, national social health insurance covered CXRs for individuals with TB symptoms or other clinical indications; those CXR costs are thus not included in the estimated project costs for that ICF subgroup, since they are not applicable.

[c] Xpert cost: $5.63/test for labor and consumable supplies, using the National TB Program and Global Fund cost norms. The National TB Program provided Xpert cartridges (funded with Global Fund support).

[d] For ACF and hybrid ACF/ICF, the initial encounter included evaluation for TB infection, thus the symptom screen and CXRs in this table evaluated for both TB disease and infection.
